# Coping With Uncertainty: Use of Contemplative Practices Amid a Pandemic

**DOI:** 10.1093/geroni/igab046.509

**Published:** 2021-12-17

**Authors:** Nirmala Lekhak, Tirth Bhatta, Tim Goler, Eva Kahana

**Affiliations:** 1 University of Nevada Las Vegas, Las vegas, Nevada, United States; 2 University of Nevada, Las Vegas, Nevada, United States; 3 Norfolk State University, Norfolk, Virginia, United States; 4 Case Western University, Case Western reserve University, Ohio, United States

## Abstract

Contemplative practices have been used as coping resources to reduce the negative influences of adverse life situations on mental health. The COVID-19 pandemic has disproportionately impacted older adults, causing immense uncertainty, stress, and anxiety. By using data from our “Coping with Pandemic” nationwide web-based survey (n=1861), we examine the utilization of practices such as meditation, prayer, and yoga across social, economic, and health status during the pandemic. Consistent with studies conducted before the pandemic, we find significantly greater utilization of meditation and yoga among women and higher educated individuals. Findings showed significantly greater usage of prayer among women and Blacks. Unlike previous studies, we documented greater usage of meditative practices among Blacks than Whites. Older adults with higher anxiety were significantly more likely to practice meditation and yoga. Our study offers much needed guidance for future intervention studies aimed at improving mental health among diverse groups of older adults.

